# *Tsc2* shapes olfactory bulb granule cell molecular and morphological characteristics

**DOI:** 10.3389/fnmol.2022.970357

**Published:** 2022-09-05

**Authors:** Victoria A. Riley, Jennie C. Holmberg, Aidan M. Sokolov, David M. Feliciano

**Affiliations:** Department of Biological Sciences, Clemson University, Clemson, SC, United States

**Keywords:** tuberous sclerosis complex (TSC), tuberous sclerosis complex 2 (TSC2), mammalian target of rapamycin (mTOR), olfactory bulb (OB), granule cell, tuberous sclerosis complex 1 (TSC1)

## Abstract

Tuberous Sclerosis Complex (TSC) is a neurodevelopmental disorder caused by mutations that inactivate *TSC1* or *TSC2*. Hamartin and tuberin are encoded by *TSC1* and *TSC2* which form a GTPase activating protein heteromer that inhibits the Rheb GTPase from activating a growth promoting protein kinase called mammalian target of rapamycin (mTOR). Growths and lesions occur in the ventricular-subventricular zone (V-SVZ), cortex, olfactory tract, and olfactory bulbs (OB) in TSC. A leading hypothesis is that mutations in inhibitory neural progenitor cells cause brain growths in TSC. OB granule cells (GCs) are GABAergic inhibitory neurons that are generated through infancy by inhibitory progenitor cells along the V-SVZ. Removal of *Tsc1* from mouse OB GCs creates cellular phenotypes seen in TSC lesions. However, the role of *Tsc2* in OB GC maturation requires clarification. Here, it is demonstrated that conditional loss of *Tsc2* alters GC development. A mosaic model of TSC was created by performing neonatal CRE recombinase electroporation into inhibitory V-SVZ progenitors yielded clusters of ectopic cytomegalic neurons with hyperactive mTOR complex 1 (mTORC1) in homozygous *Tsc2* mutant but not heterozygous or wild type mice. Similarly, homozygous *Tsc2* mutant GC morphology was altered at postnatal days 30 and 60. *Tsc2* mutant GCs had hypertrophic dendritic arbors that were established by postnatal day 30. In contrast, loss of *Tsc2* from mature GCs had negligible effects on mTORC1, soma size, and dendrite arborization. OB transcriptome profiling revealed a network of significantly differentially expressed genes following loss of *Tsc2* during development that altered neural circuitry. These results demonstrate that *Tsc2* has a critical role in regulating neural development and shapes inhibitory GC molecular and morphological characteristics.

## Introduction

Tuberous Sclerosis Complex (TSC) is a genetic disorder affecting ~1 million patients worldwide and is caused by inactivating mutations in *TSC1* or *TSC2* (Osborne et al., 1991; The European Chromosome 16 Tuberous Sclerosis Consortium, 1993; Van Slegtenhorst et al., 1997; O’Callaghan et al., 1998; Feliciano et al., 2013a; Feliciano, 2020; Northrup et al., 2021; Strzelczyk et al., 2021). Growths called subependymal nodules (SENs) occur along the ventricular-subventricular zone (V-SVZ), also referred to as the subependymal zone (SEZ), in >85% of patients (Northrup et al., 1993; Adriaensen et al., 2009; Roth et al., 2013; Chan et al., 2018; Hasbani and Crino, 2018; Jansen et al., 2019). SENs are slow growing lesions that protrude into the lateral ventricles often located near the caudate nucleus and the Foramen of Monro (Nabbout et al., 1999; Jóźwiak et al., 2013). SENs can arise embryonically but grow during the neonatal period (Northrup et al., 1993, 2021; Krueger et al., 2013; Kingswood et al., 2017; Chan et al., 2018). Approximately 5%–20% of SENs become subependymal giant cell astrocytomas (SEGAs), a slow growing grade I neoplasm (Adriaensen et al., 2009; Northrup et al., 2013; Chan et al., 2018). TSC patients also have olfactory tract lesions and olfactory bulb (OB) hamartomas (de León et al., 1988). Rhinencephalon lesions including OB aplasia, OB hypoplasia, and OB hamartomas can be identified by magnetic resonance imaging (Manara et al., 2018).

The rhinencephalon abnormalities in TSC patients correlate with the presence of growths called cortical tubers (Manara et al., 2018). Cortical tubers cause medically refractory seizures and tuber resection can ameliorate epilepsy in TSC patients (Feliciano, 2020). The origins of tubers are unclear and likely arise embryonically, however recent evidence supports that *TSC1* or *TSC2* mutations in caudal late inhibitory neuroprogenitors surrounding the lateral ventricles can cause SENs, SEGAs, and tubers (Eichmüller et al., 2022). Indeed, deletion of *Tsc1* from medial, lateral, and caudal ganglionic eminence neural stem cells (NSCs) perturbs GABAergic cortical inhibitory neuron (CIN) development leading to delayed growth and premature death of mice (Fu et al., 2012). In this model, CINs are ectopically positioned, have hyperactive mTOR complex 1 (mTORC1), are enlarged, and have CIN subtype selective changes, reducing the number of neuropeptide Y and calretinin but not somatostatin or parvalbumin CINs (Fu et al., 2012). In a complementary model, loss of *Tsc1* caused somatostatin positive CINs to gain parvalbumin CIN characteristics including increased mTOR activity, expression of K_v_3.1, and acquisition of fast spiking properties (Malik et al., 2019). Moreover, loss of *Tsc1* alters CIN morphology and connectivity leading to social behavior defects that are sensitive to mTOR inhibitors between the third and fourth postnatal weeks (Amegandjin et al., 2021). Thus, loss of function mutations in TSC genes affected inhibitory neuron characteristics which may have relevance for understanding epileptogenesis and neuropsychiatric manifestations in TSC.

OB granule cells (GCs) are inhibitory interneurons produced throughout infancy by subventricular zone (SVZ) NSCs (Sanai et al., 2011). SVZ NSCs generate transit amplifying progenitor cells that produce neuroblasts (Lim and Alvarez-Buylla, 2016). Neuroblasts migrate rostrally by way of the rostral migratory stream (RMS) into the OB (Whitman and Greer, 2009). Incidentally, humans have a phylogenetically unique bifurcation of the RMS, the medial migratory stream, that siphons neuroblasts toward the ventromedial prefrontal cortex (Sanai et al., 2011). Once within the OB, neuroblasts predominantly mature into GCs (Whitman and Greer, 2009; Lim and Alvarez-Buylla, 2016). Given the relationship between SVZ NSCs and OB GCs, a hypothesis is that SVZ NSC *TSC1/TSC2* mutations cause abnormal OB development (Feliciano et al., 2013a). Previous studies examined the role of TSC genes in OB development by deletion of *Tsc1* in neonatal SVZ NSCs (Zhou et al., 2011; Feliciano et al., 2012). Conditional *Tsc1* deletion caused neuroblast migration defects and fewer OB GCs (Zhou et al., 2011). Neuroblasts lacking *Tsc1* migrated slower, and GCs were disorganized, had hyperactive mTORC1, and were cytomegalic (Feliciano et al., 2012). *Tsc1* regulation of GC morphology was further confirmed to include a reduction in perisomatic bouton density and a reduction in GC spine density (Amegandjin et al., 2021). Given that the *Tsc1* and *Tsc2* encoded proteins hamartin and tuberin form a GTPase activating protein complex that inhibits Rheb activation of mTOR, it is not surprising that Rheb electroporation can phenocopy the effects of *Tsc1* knockout (Lafourcade et al., 2013; Sokolov et al., 2020). Moreover, loss of mTOR or upstream amino acid transporters required for mTORC1 activation reduced GC soma size and dendrite arborization (Skalecka et al., 2016; Sokolov et al., 2020). Taken together, these results provide compelling evidence that the mTOR pathway controls inhibitory neuron characteristics. Despite the evidence that the mTOR pathway is required for OB GC development, the role of *Tsc2* in GC development has not been fully elucidated. Understanding the contribution of *Tsc2* to development is critically important since patient *TSC2* mutations are associated with more severe neurological sequalae.

Here, the role of *Tsc2* in OB GC development, maturation, and maintenance was evaluated.

## Materials and Methods

### Animals

Clemson University Institutional Animal Care and Use Committee approved all performed experiments, and all guidelines set forth by the Clemson University Institutional Animal Care and Use Committee and were compliant with the Animal Care and Use Review Office (ACURO), a component of the USAMRDC Office of Research Protections (ORP) within the Department of Defense (DoD). (B6.Cg-*Gt(ROSA)26Sor^tm9(CAG-tdTomato)Hze^*^/J^; Strain #007909), C57BL/6-Tg^(Nes-cre/ERT2)KEisc/J^ (Strain #:016261), Tsc2^tm1.1Mjg/J^ (Strain #027458) were acquired from Jackson Laboratories. Mice were housed under pathogen-free conditions with a 12-h light/dark cycle and fed *ad libitum*.

### Electroporation

Neonatal mice were electroporated as previously described. Mice (P0-P1) were injected with equal concentrations and volumes of DNA plasmids (~1.5–2.0 μg/μl) diluted in phosphate buffered saline (PBS) with 0.1% fast green and includes the following plasmids: CAG-CRE (Plasmid #13775), CAG-GFP (Plasmid #11150), CAG-CRE-ERT2 (Plasmid #13777), and CAG-tdTomato (Addgene #83029; Matsuda and Cepko, 2007). DNA was injected into the lateral ventricles illuminated with a fiber optic light source and delivered using a borosilicate glass micropipette generated from pulled capillary tubes. Borosilicate capillary tubes were pulled with a P97 Sutter micropipette puller. Tweezer electrodes (model 520; BTX) were rinsed in 0.9% saline solution and placed swept over the head of neonatal pups using five, 100-volt square pulses of 50 ms duration with 950-ms intervals were applied using a pulse generator (ECM830; BTX).

### Genotyping PCR

Tissue was incubated in 50 mM NaOH and 0.2 mM EDTA at 50°C for 90 min. An equal volume of 100 mM Tris-HCl was added to samples. A PCR master mix was generated by mixing water, buffer, dNTPs, primers and Taq DNA Polymerase and adding to individual 50 μl PCR tubes. Samples were gently vortexed and centrifuged. Thin-walled PCR tubes are placed on ice and the following components are combined: 10× Taq Buffer dNTP Mix, 2 mM each (#R0241), forward primer 0.1–1.0 μM and reverse primer 0.1–1.0 μM, 1 mM MgCl_2_, template DNA (~10 pg), 1.25 U of Taq DNA Polymerase, and nuclease-free water (#R0581) for a final volume of 50 μl. Samples were vortexed and spun down. PCR was performed with the following conditions: Initial denaturation step at 95°C for 3 min, followed by 32 cycles of denaturation at 95°C for 30 s, an annealing step at 60°C for 30 s, and an extension at 72°C for 30 s followed by a final extension at 72°C for 5 min. Mice having conditional *Tsc2* alleles are distinguished by endpoint genotyping PCR using the following primer sequences, 5’-ACAATGGGAGGCACATTACC-3’ and 5’AGCAGCAGGTCTGCAGTG-3’. A wild type amplicon of 191 bp and a floxed amplicon of 250 bp are produced. Tomato genes were identified by endpoint genotyping PCR using the following primer sequences, 5’-AAGGGAGCTGCAGTGGAG TA-3’ and 5’-CCG AAAATCTGTGGGAAG TC-3’ and 5’-GGCATTAAAGCAGCGTATCC-3’ and 5’-CTGTTCCTGTACGGCATGG-3’ generating a tdTomato Mutant ~200 bp amplicon or a 297 bp Wild type amplicon. Nestin-CRE-ER^T2^ mice were genotyped with one of two primer sets 5’-ATGCAGGCAAATTTTGGTGT-3’ (CRE) and 5’-CGCCGCTACTTCTTTTCA AC-3’ (Nestin) and 5’-AGTGGCCTCTTCCAGAAATG-3’ (Internal Positive Control) and 5’-TGCGACTGTGTCTGATTTCC-3’ (Internal Positive Control). Alternatively, Nestin-CRE-ER^T2^ mice were genotyped with 5’-ATACCGGAGATCATGCAAGC-3’ (CRE) and 5’-GGCCAGGCTGTTCTTCTTAG-3’ (ER^T2^) and 5’-CTAGGCCAAGAATTGAAAGATCT-3’ (Internal Positive Control) and 5’-GTAGGTGGAAATTCTAGCATCATCC-3’ (Internal Positive Control).

### Long range PCR

DNA was isolated using the Qiagen DNeasy Blood and Tissue Kit (250; Catalog #69506). Samples were placed into microcentrifuge tubes with buffer ATL and Proteinase K and were incubated at 56°C and vortexed every hr until tissue was digested. Buffer AL and ethanol were added to digested tissues. The mixture was then pipetted into a DNeasy mini spin column and centrifuged at room temperature at 6,000× *g* for 1 min. The column is then placed into a new collection tube with AW1 and centrifuged at 6,000× *g* for 1 min. The collection tube is replaced, buffer AW2 is added to the sample, and centrifuged at 20,000× *g* for 3 min. The eluant is removed and the sample is centrifuged for 3 min at 2,000× *g* . The DNeasy mini spin column is then placed into a microcentrifuge tube and 50 μl of buffer AE is pipetted directly onto the membrane. The samples were incubated at room temperature for 5 min and subsequently centrifuged at 6,000× *g* for 1 min. Eluted sample is placed onto the DNeasy mini spin column and centrifuged again at 6,000× *g* for 1 min. Samples were quantified with a NanoDrop spectrophotometer. 1 μl of DNA was mixed with master mix that was generated with a Qiagen Long Range PCR Kit. Master mix consisted of 5 μl 10× Long Range PCR Buffer with Mg^2+^, 2.5 μl 10 mM dNTP mix, 10 μl 5× Q Solution, 0.2 μl of each primer, 0.4 μl LongRange PCR Enzyme Mix, and RNase free water to 50 μl total per reaction. Forty-nine microliter of master mix was added to a PCR tube along with 1 μl of DNA. The samples were then placed into a thermocycler and subjected to the following protocol; Initial activation step at 93°C for 3 min, followed by 32 cycles of the following 3-steps; Denaturation at 93°C for 15 s, an annealing step at 62°C for 30 s, and an extension at 68°C for 1 min 30 s followed by a final extension at 72°C for 5 min. Samples were loaded onto a 2% agarose gel with 1× Blue Juice and run at 100 V for 20–30 min. The primers for detecting Tsc2 wild-type, floxed, and recombined alleles are: P3F 5’-AAGATTCCGGCTTGAAGGAG-3’, P4F 5’-CACTA-GTCTAGCCTGACTCT-3’, and P3R 5’-GAGGACAAGCCAACATCCAT-3’.

### Immunohistochemistry

Pentobarbital (50 mg/kg) administered by intraperitoneal injection was used to sedate mice prior to decapitation. Brains were dissected in room temperature PBS, transferred to 4% paraformaldehyde (in PBS), and incubated overnight at 4°C. Brains were rinsed in PBS and mounted in low melt agarose (3%). A Leica VTS 1000 vibratome was used to slice brains coronally in 300 μm sections. Sections were blocked in PBS containing 0.1% Triton X-100, 0.1% Tween-20 and 2% BSA for 1 h at room temperature. Sections were washed in PBS containing 0.1% Tween-20 three times. Sections were incubated in primary antibody pS6 (rabbit anti-pS6; 1:1,000; Cell Signaling Technology; Ser 240/244, 61H9, #4838) overnight at 4°C. After three washes additional washes in PBS containing 0.1% Tween-20, slices were incubated with the appropriate secondary antibody (Alexa Fluor series; 1:500; Invitrogen) overnight at 4°C. Sections were mounted in ProLong Gold Antifade Mountant (ThermoFisher). Each staining was replicated on 4–13 mice per condition. Images were acquired on a spectral confocal microscope (Leica SPE) with a ×20 dry objective (N.A. 0.80). Low-magnification images were acquired with a ×5 dry (N.A. 0.15) objective.

### Image analysis

Images (×20) were uploaded and analyzed using FIJI (ImageJ 1.5 g). Simple neurite tracer plug-in was used to trace dendrite processes of RFP positive cells from 4 to 11 mice per condition. Sholl analysis was performed at 10 μm intervals to quantify both apical and basal dendritic arborization using the Sholl plug-in. A total number of dendritic crossings were calculated by taking the sum of crossings at 10 μm intervals for each traced neuron and averaging the total number of crossings per neuron in each condition for 4–11 mice.

Images (×20) of RFP positive OB GCs stained by immunohistochemistry were uploaded to FIJI (ImageJ 1.5 g). The freehand selection tool was used to trace a region of interest (ROI) on electroporated and non-electroporated cells in the same Z section was performed and a mean gray value to quantify the staining intensity of pS6 was recorded. Ratios of staining in electroporated and non-electroporated cells were compared performed for RFP positive cells in Tsc2^wt/wt^, Tsc2^wt/f^, and Tsc2^f/f^ conditions.

Images were uploaded to FIJI and subject to a custom macro available with supplemental data. Briefly, results were cleared, channels were split, and a maximum Z projection of RFP was made. Automated thresholding using Renyi Entropy was performed with images inverted and resulting binary image subjected to binary watershed. Resulting binary images were subject to particle analysis using custom settings (size = 20–400 circularity = 0.5–1.00) to only capture somas and the size and number of particles were saved along with drawing of particles. The size of particles was used for quantification of soma size. The drawing of particles was subsequently subjected to processing in geological image analysis software as previously described (GIAS v1.12; Feliciano et al., 2012). Default settings were applied, and the nearest neighbor distance Ra was calculated. Organization is then measured as the theoretical Poisson distribution in relation to the experimental distribution.

### RNA sequencing and bioinformatics

OBs (two for each mouse, from three mice each condition) were collected and homogenized in 500 μl TRIzol reagent (ThermoFisher). Samples were incubated for 5 min at 4°C and 100 μl chloroform was subsequently added. Samples were centrifuged for 15 min at 12,000× *g* at 4°C and the aqueous layer was collected and transferred to a 1.5 ml centrifuge tube. 250 μl of isopropanol was added to samples and incubated for ten minutes at 4°C. The samples were centrifuged for ten minutes at 12,000× *g* at 4°C and the supernatant discarded. Pellets were resuspended in 500 μl 75% ethanol and vortexed before being centrifuged for 5 min at 7,500× *g* at 4°C. Pellets were allowed to dry and rehydrated in 35 μl of RNase free ultrapure water. Samples were incubated at 60°C for 15 min to complete RNA isolation. RNA concentrations and purity were assessed using a NanoDrop Lite Spectrophotometer (Thermo Scientific). Sample quantity and RIN values were determined.

RNA concentration and quality was verified by Qubit assay and Agilent Tapestation analysis which confirmed RNA integrity. Libraries were prepared using NEBNext Ultra RNA Library Preparation Kit according to the manufacturer’s protocol (New England Biolabs). Samples were sequenced using 150 base pair end reads on an Illumina HiSeq. Read adapter sequences and nucleotides with poor quality were trimmed using Trimmomatic 0.36 and mapped to the Mus musculus GRCm38 reference genome available on ENSEMBL using the STAR aligner. 98.28% of reads were mapped with an average quality Q score of 35.69. 402,490,214 reads over the six samples had an average of 66,000,935 reads per sample. RNA sequencing aligner was executed using splicing aligner. The subread package was used to calculate unique exon hit counts. Unique exon hit counts were analyzed using differential expression analysis (DESeq2) and differential splice variant expression (DEXseq) analysis. Correlation between samples and treatment was performed by PCA plot. A heatmap of the top 30 genes based on p-adjusted value were subject to bi-cluster analysis to check for correlation between samples. Gene ontology analysis was performed by implementing GeneSCF, gene networks were identified by Cytoscape 3.60 using Genemania, and SynGO analysis was performed as we have previously described (Morton et al., 2018; Koopmans et al., 2019).

### Statistics

Data were graphed and analyzed with GraphPad Prism software (Version 8.2.0, GraphPad Software Inc.). Statistical significance was determined by Student’s t-test (tamoxifen experiments), one-way analysis of variance (ANOVA; ROI and total number of crossings analysis) with multiple comparisons test, or two-way ANOVA with Tukey’s multiple comparisons test (Sholl analysis). All experiments were performed on 4–11 mice per condition per time point. N (number of mice) and n (number of cells) are listed where applicable. Error bars are reported as standard error mean.

## Results

### Modeling loss of Tsc2 in OB granule cells

**Figure 1:**
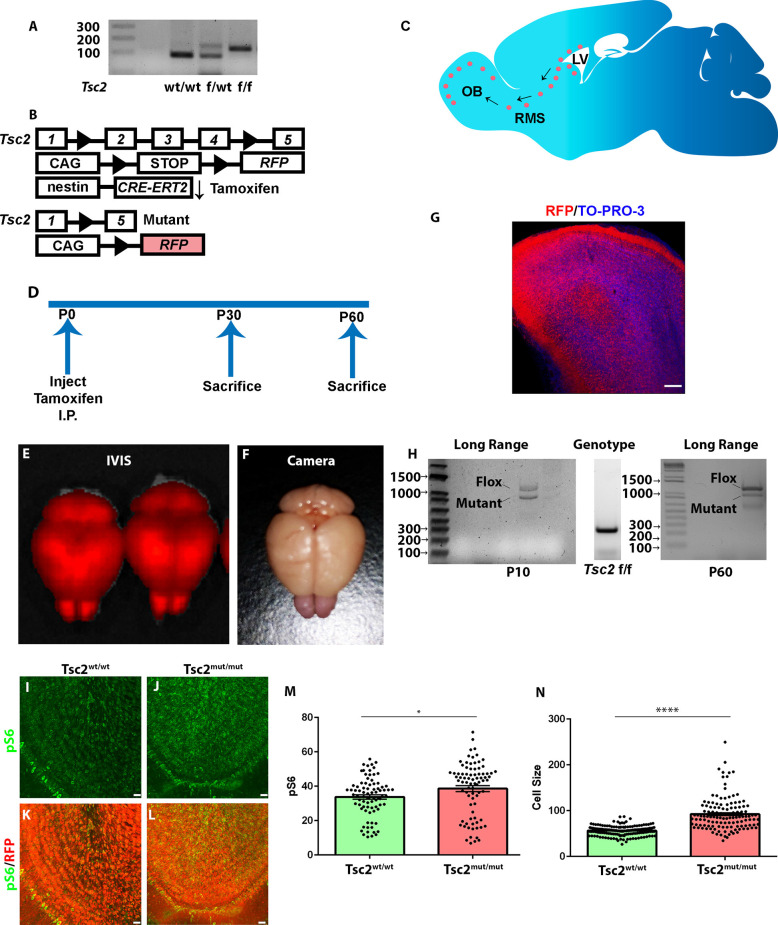
Modeling loss of Tsc2 in OB granule cells. **(A)** Genotyping PCR indicating TSC genotypes. **(B)** Genotypes of mice demonstrating that mouse NSCs express a tamoxifen inducible form of the CRE recombinase. Upon application of this exogenous ligand, CRE recombines regions flanked by loxP sites. This leads to Tsc2 mutation and induction of RFP. **(C)** Sagittal section of the mouse brain depicting the presence of NSCs around the lateral ventricle that produce neuroblasts that migrate into the RMS and enter the OB. **(D)** Time course for experimentation. **(E)** Whole brain fluorescent imaging. **(F)** Photograph showing pink OBs. **(G)** Confocal image of sagittal section of a P60 OB. **(H)** PCR demonstrating that the *Tsc2* gene undergoes recombination. **(I–L)** Confocal image of OBs demonstrating OB pS6 levels. **(M)** Quantification of OB pS6. **(N)** Quantification of cell size. ^*^p < 0.05, ^****^p < 0.0001; OB, olfactory bulb; RMS, rostral migratory stream; LV, lateral ventricle.

*Tsc1* and *Tsc2* genes encode for the proteins hamartin and tuberin, respectively. Hamartin and Tuberin form a GTPase activating protein heteromer that prevents Rheb activation of mTOR. Thus, inactivating mutations in *TSC1* or *TSC2* increase mTOR activity. Mice having a conditional *Tsc2* allele (*Tsc2^tm1.1Mjg^*/J) containing *loxP* sites flanking exons 2, 3, and 4 (henceforth referred to as *Tsc2*^f/f^) detectable by PCR were crossed to Ai9 mice (B6.Cg-*Gt(ROSA)26Sor^tm9(CAG-tdTomato)Hze^*/J) that have a *loxP-*flanked STOP cassette that prevents transcription of a CAG-promoter driven variant of red fluorescent protein (tdTomato, RFP; Figures 1A,B). These mice were further crossed to *Nestin*-CRE-ER^T2^ mice (Figure 1B). Nestin mice express a Cre recombinase fused to an estrogen receptor (ER) mutant which does not bind the physiological ligand, 17β-estradiol (Lagace et al., 2007). The fusion protein is downstream of the *Nestin* promoter (Lagace et al., 2007). Application of the exogenous ligand 4-hydroxytamoxifen causes cytoplasmic localized CRE to dimerize and enter the nuclear compartment where it subsequently excises regions of DNA flanked by *loxP* sites. The *Nestin* promoter is active in NSCs and CRE-ER^T2^ will therefore remove a stop sequence that induces RFP expression and *Tsc2* recombination along the lateral ventricle (Figures 1B,C; Lagace et al., 2007). Genomic recombination subsequently causes recombination in NSC progenitors, neuroblasts along the RMS, and neurons within the OB (Figures 1B,C).

4-hydroxytamoxifen was injected at postnatal day (P)2–3 into *Tsc2*^f/f^ mice to evaluate the role of *Tsc2* in GC development. Mice were sacrificed at P10, P30, or P60, brains were removed, and subjected to IVIS imaging (Figure 1D). IVIS imaging confirmed robust recombination within the cerebellum, cortex, and OB (Figure 1E). RFP expression could be detected in whole brain photographs and appeared as a pinkish hue that was especially apparent within the OB (Figure 1F). OB RFP expression was confirmed by confocal microscopy (Figure 1G). Long-range PCR of *Tsc2*^f/f^ OBs confirmed the presence of the conditional allele (1,367 base pairs) that had undergone genomic recombination to form *Tsc2*^mutant (mut)^ (1,090 base pairs) alleles at P10 in the OB and this was further confirmed at P60 even though genotyping PCR confirmed the presence of only floxed *Tsc2* alleles in tail samples (Figure 1H). While the presence of a recombined allele further confirmed CRE functionality, the presence of non-recombined alleles could be caused by multiple explanations. One reason for the presence of non-recombined alleles is that mitral and tufted cells, astrocytes, or earlier born granule cells contain floxed alleles but are not targeted for recombination. Alternatively, incomplete activation of *Nestin*-CRE-ER^T2^ might fail to recombine alleles in granule cells. Another possibility is that loss of only one allele occurs within individual cells. We sought to perform immunohistochemical analysis that would approximate *Tsc2* activity to distinguish between these possibilities. Tuberin is the GTPase activating domain of a heterotrimeric complex that also contains hamartin encoded by *Tsc1* and TBC1D7 that inhibit the Rheb GTPase from activating the protein kinase mTOR. Thus, inactivating mutations in *TSC2* or *TSC1* activate mTOR. mTOR complex 1 (mTORC1) phosphorylation of the ribosomal S6 subunit was therefore examined. Tsc2^mut/mut^ GCs had a ~5% increase in pS6 at P10 (data not shown). *Tsc2* mutation increased phospho-S6 (240/244) levels by P30 according to automated analysis of individual z-sections (*Tsc2*^wt/wt^; mean = 33.74 ± 1.268 *n* = 81 images vs. *Tsc2*^mut/mut^; mean = 38.63 ± 1.720, *n* = 82 images; *p* = 0.0236; Figures 1I–M) and of Z-projected images as expected (*Tsc2*^wt/wt^; mean = 50.19 ± 2.780 *N* = 9 mice vs. *Tsc2*^mut/mut^; mean = 89.91 ± 8.360, *n* = 8 mice; *p* = 0.0003; data not shown). mTORC1 stimulates 5’ TOP mRNA translation leading to anabolic cell growth. As further confirmation of recombination, cell size was increased following loss of *Tsc2* (*Tsc2*^wt/wt^; mean = 56.47 ± 0.8288, *n* = 163 cells vs. *Tsc2*^mut/mut^; mean = 92.31 ± 3.146, *n* = 129 cells; *p* < 0.0001; Figure 1N). Taken together, loss of *Tsc2* activates the mTORC1 pathway in OB GCs.

### Electroporation models somatic Tsc2 mutation

**Figure 2:**
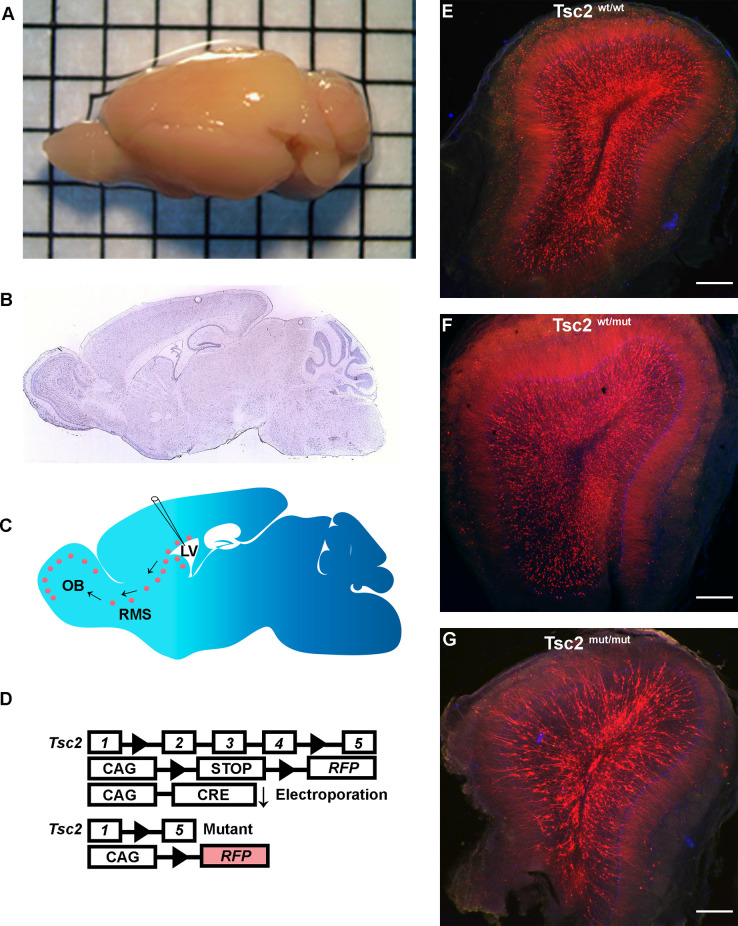
Electroporation models somatic Tsc2 mutation. **(A)** Image of mouse brain demonstrating an electroporated brain. **(B)** Sagittal section of mouse brain (Allen Brain Institute). **(C)** Schematic of electroporation demonstrating labeling of NSCs, neuroblasts, and GCs. **(D)** Schematic diagram of conditional Tsc2 alleles and inducible RFP before and after electroporation of CRE recombination. **(E–G)** 5x images of coronal sections of OBs.

Since tamoxifen-induced recombination robustly labeled GCs with RFP, overlap of the neuropil from adjacent GCs prevented morphological analysis of dendrites. Neonatal electroporation was employed by injecting CRE and/or fluorescent protein plasmids into the lateral ventricles of mice having conditional *Tsc2* alleles to circumvent this limitation (Figures 2A–D). Electroporated plasmid DNA is taken up by dividing NSCs and passed to NSC progeny. Therefore, GFP positive GCs have approximately similar birthdates during the first 7–10 days post-electroporation. Moreover, RFP is permanently expressed in recombined cells and all progeny including OB GCs irrespective of the presence of GFP. Finally, non-electroporated cells will not lose *Tsc2* thereby producing a mosaic pattern of mutation as is proposed to occur in TSC and highlighted distinct differences between *Tsc2*^wt/wt^, *Tsc2*^f/wt^, and *Tsc2*^f/f^ conditions (Figures 2E–G).

### OB GC organization following loss of Tsc2

**Figure 3:**
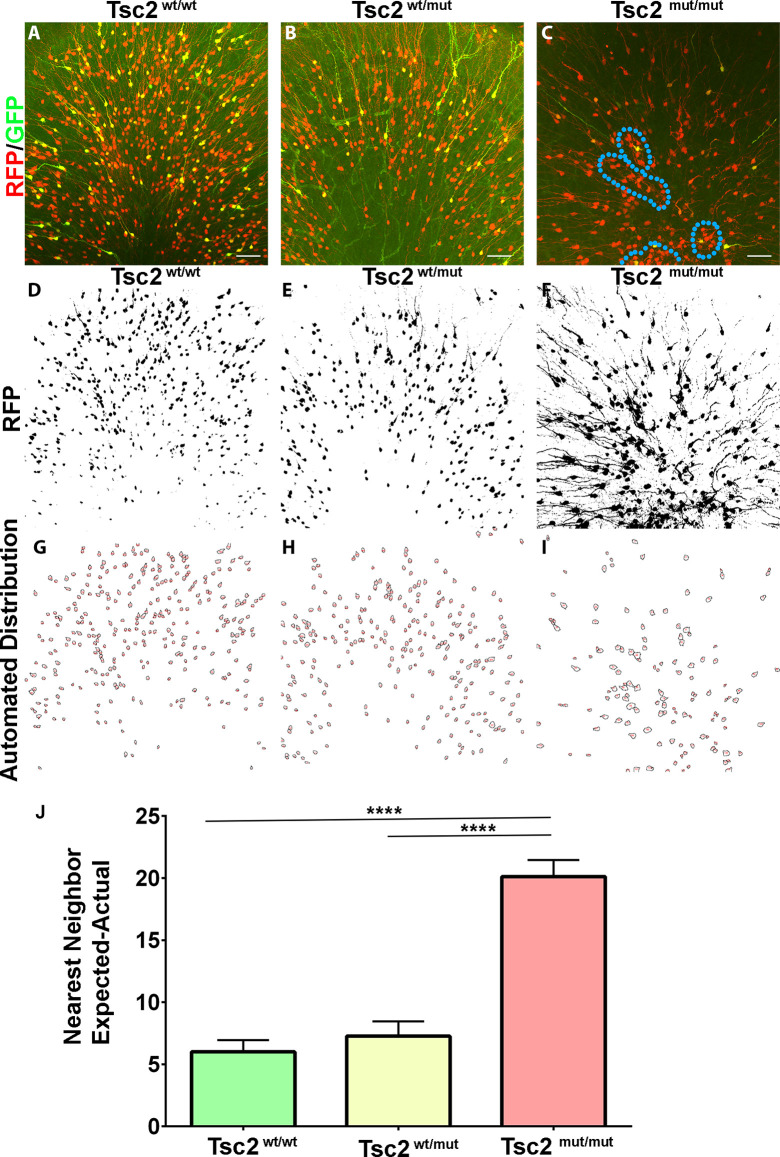
OB GC organization following loss of Tsc2. **(A–C)** 20× confocal images of coronal sections of OB GCs labeled with GFP (green) demonstrating recombination and expression of RFP (red), and pS6 staining (blue). **(D–F)** Z projections of RFP and automated thresholding. **(G–I)** Automated drawing of GC somas. **(J)** Quantification of GC organization based on expected nearest neighbor calculations in relation to actual calculations. Blue dotted line outlines clusters of ectopic cytomegalic neurons. ****p < 0.0001.

Chief among differences seen at low magnifications was the organization of *Tsc2*^mut/mut^ OBs in comparison to *Tsc2*^wt/wt^ and *Tsc2*^f/wt^ OBs. Higher magnification images of the granule cell layer were subsequently, taken (Figures 3A–C). Images were subjected to custom macros that generated Z projections of RFP positive recombined cells and created binary images (Figures 3D–F). The macros removed dendrites and outlined GC somatas (Figures 3G–I). Automated distribution analysis based on nearest neighbor calculations were determined. *Tsc2*^mut/mut^ theoretical cell distributions were greatly different from *Tsc2*^wt/wt^ and *Tsc2*^mut/wt^ conditions (*Tsc2*^wt/wt^, *N* = 4, mean = 6.009 ± 0.9435, vs. *Tsc2^wt^*^/mut^, *N* = 4, mean = 7.269 ± 1.187 vs. *Tsc2*^mut/mut^, *N* = 4, mean = 20.12 ± 1.326, *n* = 129 cells; *p* < 0.0001; Figure 3J). Taken together, the loss of both *Tsc2* copies led to changes in the organization of the OB, with an increased presence of ectopic clusters of cytomegalic neurons.

### Loss of Tsc2 in OB granule cells activates the mTOR pathway

**Figure 4:**
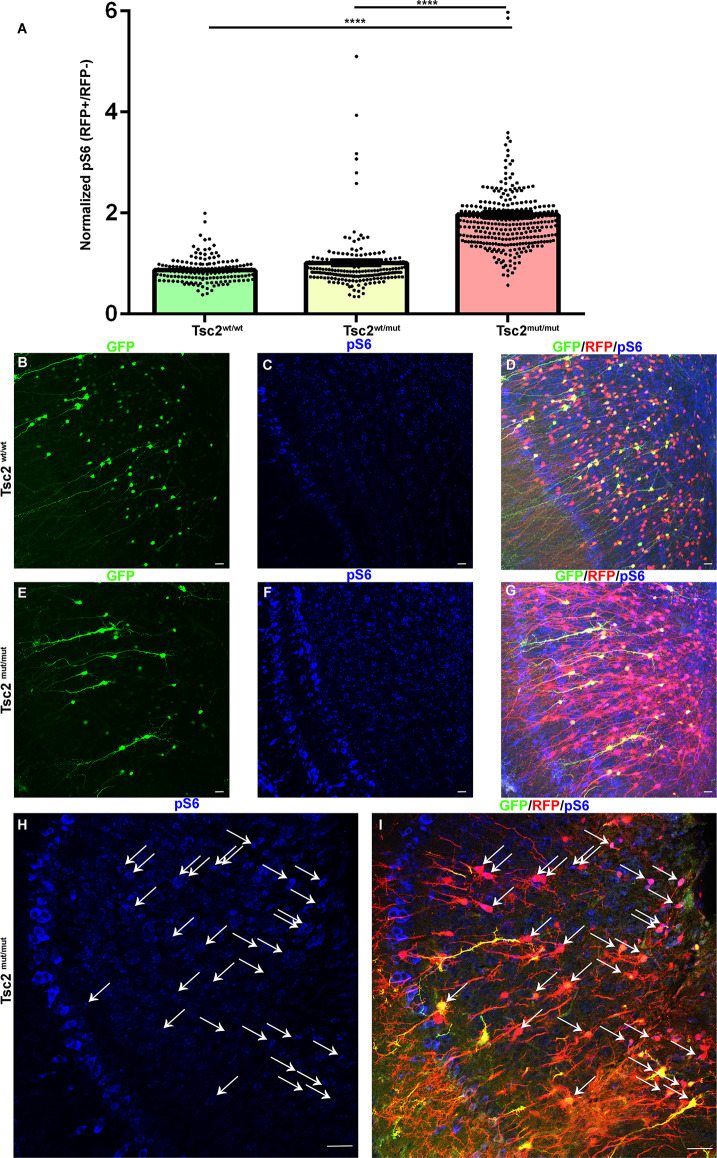
Loss of Tsc2 in OB granule cells activates the mTOR pathway. **(A)** Quantification of relative pS6 levels. **(B–I)** 20× confocal images of coronal sections of OB GCs labeled with GFP (green) demonstrating recombination and expression of RFP (red), and pS6 staining (blue). Arrows indicate Tsc2^mut/mut^ GCs with elevated pS6. ****p < 0.0001.

mTOR activity was examined in OB GCs subjected to CRE and GFP electroporation. Although large mitral and tufted cells have high levels of pS6, P30 OB GCs had detectable pS6 that was prevalent within somas and the primary dendrites as highlighted by GFP as previously observed. pS6 levels were measured and compared to neighbor non-electroporated cells in the granule cell layer as an internal normalization for *Tsc2*^mut/mut^, *Tsc2*^wt/wt^, and *Tsc2*^wt/mut^ GCs. *Tsc2*^wt/wt^ and *Tsc2*^wt/mut^ GCs pS6 levels did not significantly differ from one another (Figure 4A). However, the mean of pS6 staining for *Tsc2*^mut/mut^ GCs increased by ~226% and 194% in comparison to *Tsc2*^wt/wt^ and *Tsc2*^wt/mut^ GCs, respectively (*Tsc2*^wt/wt^, *n* = 155 cells, *N* = 7 mice, Mean =0.8680 ± 0.01966, *Tsc2*^wt/mut^ *n* = 212, *N* = 8 mice, Mean = 1.013 ± 0.05985, *Tsc2*^mut/mut^ *n* = 301, *N* = 9 mice, Mean = 1.965 ± 0.07024; *P* < 0.0001, *P* < 0.0001; Figure 4A). Notably, *Tsc2*^mut/mut^ cell pS6 levels appeared elevated in *Tsc2*^mut/mut^ GCs and appeared larger than *Tsc2*^wt/wt^ and *Tsc2*^wt/mut^ (Figures 4B–I). The observation of hyperactive mTORC1 signaling in neurons is also consistent with the appearance of TSC neurological malformations, including in tubers. Taken together, loss of two alleles of *Tsc2* activates mTORC1.

### Loss of Tsc2 causes GC cytomegaly

**Figure 5:**
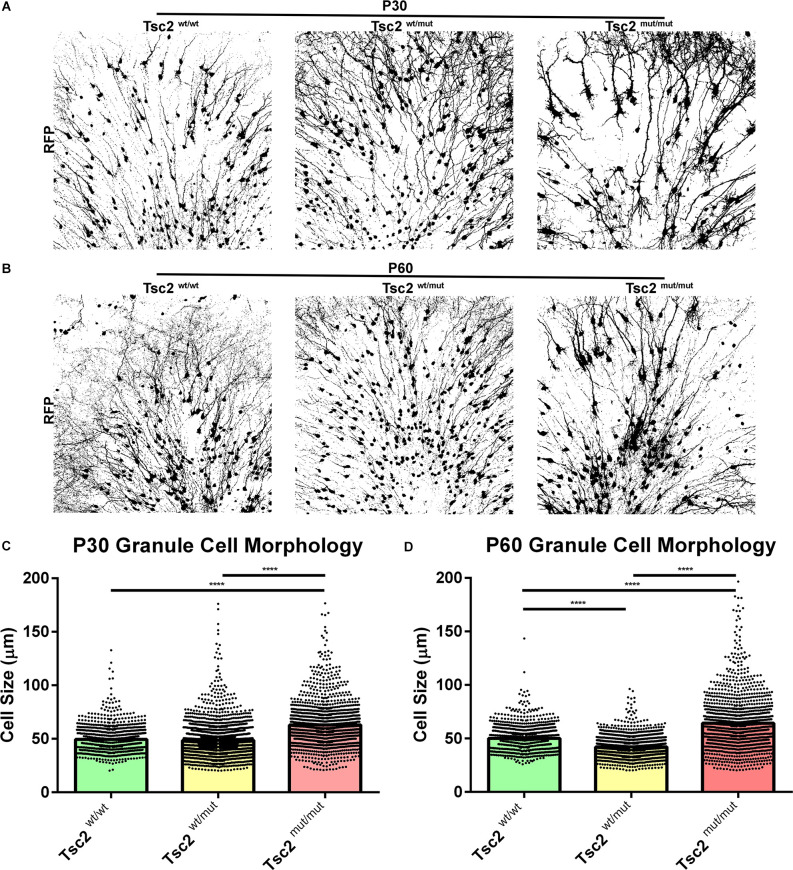
Loss of Tsc2 causes GC cytomegaly. **(A)** P30 OB GC Z projection converted to binary. **(B)** P60 OB GC Z projection converted to binary. **(C)** Quantification of cell size at P30. **(D)** Quantification of cell size at P60. ****p < 0.0001.

mTOR regulates anabolic growth. Loss of *Tsc2* is therefore predicted to increase cell size as we noted in previous images (Figure 4). Z projected images subjected to automated thresholding depicted gross morphological differences between *Tsc2*^mut/mut^, *Tsc2*^wt/wt^, and *Tsc2*^wt/mut^ GCs at P30 (Figure 5A) and P60 (Figure 5B). Notably, soma sizes appeared increased, the gauge of dendrites appeared thickened, and dendrite arbors appeared more prominently. Soma size analysis identified that *Tsc2^mut/mut^* cells were 27% and 30% larger than *Tsc2*^wt/wt^ GCs and *Tsc2*^wt/mut^ GCs, respectively (*Tsc2*^wt/wt^, *n* = 1,158 cells, *N* = 8 mice, Mean = 49.41 ± 0.3360, *Tsc2*^wt/mut^, *n* = 2,497, *N* = 10 mice, Mean = 48.51 ± 0.2921, *Tsc2*^mut/mut^, *n* = 1,426, *N* = 13 mice, Mean = 62.92 ± 0.5401; *P* < 0.0001, *P* < 0.0001; Figure 5C). *Tsc2*^wt/wt^ GCs did not significantly differ from *Tsc2*^wt/mut^ confirming that loss of two *Tsc2* alleles is necessary to alter soma size. Soma size at P60 was further examined to determine the extent that GCs continue to become enlarged. Somas of *Tsc2^mut/mut^* GCs were larger than *Tsc2*^wt/wt^ GCs and *Tsc2*^wt/mut^ GCs, respectively (*Tsc2*^wt/wt^, *n* = 1,100 cells, *N* = 4 mice, Mean = 50.04 ± 0.3389, *Tsc2*^wt/mut^ *n* = 1718, *N* = 7 mice, Mean = 42.13 ± 0.2234, *Tsc2*^mut/mut^ *n* = 1,229, *N* = 4 mice, Mean = 64.31 ± 0.7314; *P* < 0.0001; Figure 5D). Taken together, *Tsc2* is a determinant of soma size, the acquisition of which is predominantly determined during the first 30 days and maintained thereafter.

### Neuron dendrite morphology is altered by loss of Tsc2

**Figure 6:**
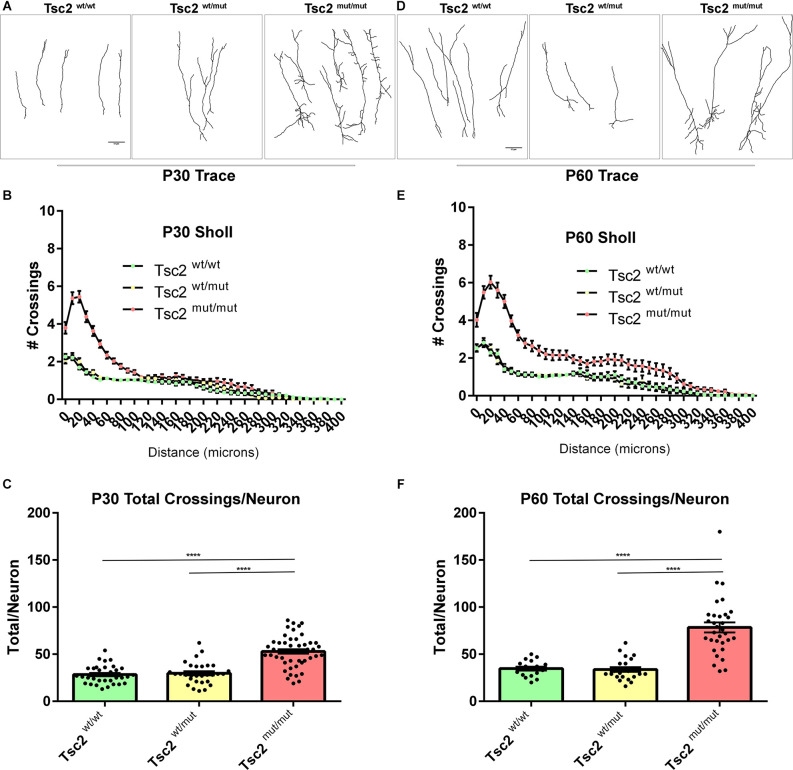
Neuron dendrite morphology is altered by loss of Tsc2. **(A)** P30 traces of OB GC dendrite arbors. **(B)** Quantification of the number of dendrite crossings of concentric circles drawn in relation to distance at P30, also known as sholl analysis. **(C)** Quantification of the total number of dendrite crossings per neuron at P30. **(D)** P60 traces of OB GC dendrite arbors. **(E)** Quantification of the number of dendrite crossings of concentric circles drawn in relation to distance at P60, also known as sholl analysis. **(F)** Quantification of the total number of dendrite crossings per neuron at P60. ****p < 0.0001.

Tsc2^mut/mut^ GCs were larger than control GCs and dendrite arbors were greatly impacted by loss of *Tsc2* (Figure 5). Dendrites were traced and sholl analysis was performed at P30 for *Tsc2*^wt/wt^, *Tsc2*^wt/mut^, and *Tsc2*^mut/mut^ GCs since OB GC dendrite arborization is dependent upon mTORC1 signaling (Figure 6A). *Tsc2*^mut/mut^ GC dendrite arbors were vastly different in comparison to *Tsc2*^wt/wt^ and *Tsc2*^wt/mut^ GCs. *Tsc2*^mut/mut^ GCs had more basal dendrites which sprouted from numerous regions of the soma, occasionally branched, and were longer. The changes in *Tsc2*^mut/mut^ GC basal dendrites led to a larger number of crossings in sholl analysis proximal to the soma (Figure 6B). Supraphysiological numbers of dendrites sprouted from apical dendrites of *Tsc2*^mut/mut^ GC projecting toward the external plexiform layer of the OB although they were similar in length. Overall, the increased number of basal dendrites and sprouting of apical dendrites was associated with a statistically significant increase in the number of dendrite crossings per neuron for *Tsc2*^mut/mut^ GCs compared to *Tsc2*^wt/wt^ and *Tsc2*^wt/mut^ GCs (*Tsc2*^wt/wt^, *n* = 33 cells, *N* = 7 mice, Mean = 28.33 ± 1.684, *Tsc2*^wt/mut^ *n* = 30, *N* = 7 mice, Mean = 29.77 ± 2.135, *Tsc2*^mut/mut^ *n* = 49, *N* = 11 mice, Mean = 52.94 ± 2.360; *P* < 0.0001, *P* < 0.0001; Figure 6C). An additional cohort of OB GCs were traced from P60 mice (Figure 6D). P60 Tsc2^mut/mut^ OB GCs were similar in that proximal basal dendrite complexity was elevated (Figure 6E). Primary apical dendrite sprouts appeared to be pruned, however, distal ramifications continued to lengthen in relation to *Tsc2*^wt/wt^ and *Tsc2*^wt/mut^ GCs. The total number of dendrite crossings per neuron increased at P60 in comparison to P30 for *Tsc2*^wt/wt^ GCs as expected (Figure 6F). Moreover, the total number of dendrite crossings per neuron significantly increased for *Tsc2*^mut/mut^ GCs compared to *Tsc2*^wt/wt^ and *Tsc2*^wt/mut^ GCs (*Tsc2*^wt/wt^, *n* = 17 cells, *N* = 4 mice, Mean = 34.71 ± 2.107, *Tsc2*^wt/mut^ *n* = 22, *N* = 7 mice, Mean = 33.68 ± 2.504, *Tsc2*^mut/mut^ *n* = 32, *N* = 9 mice, Mean = 78.38 ± 5.302; *P* < 0.0001, *P* < 0.0001). Taken together, developmental loss of *Tsc2* caused molecular, biochemical, and morphological changes in P60 GCs.

### Loss of Tsc2 in mature GCs has modest effects on morphology

**Figure 7:**
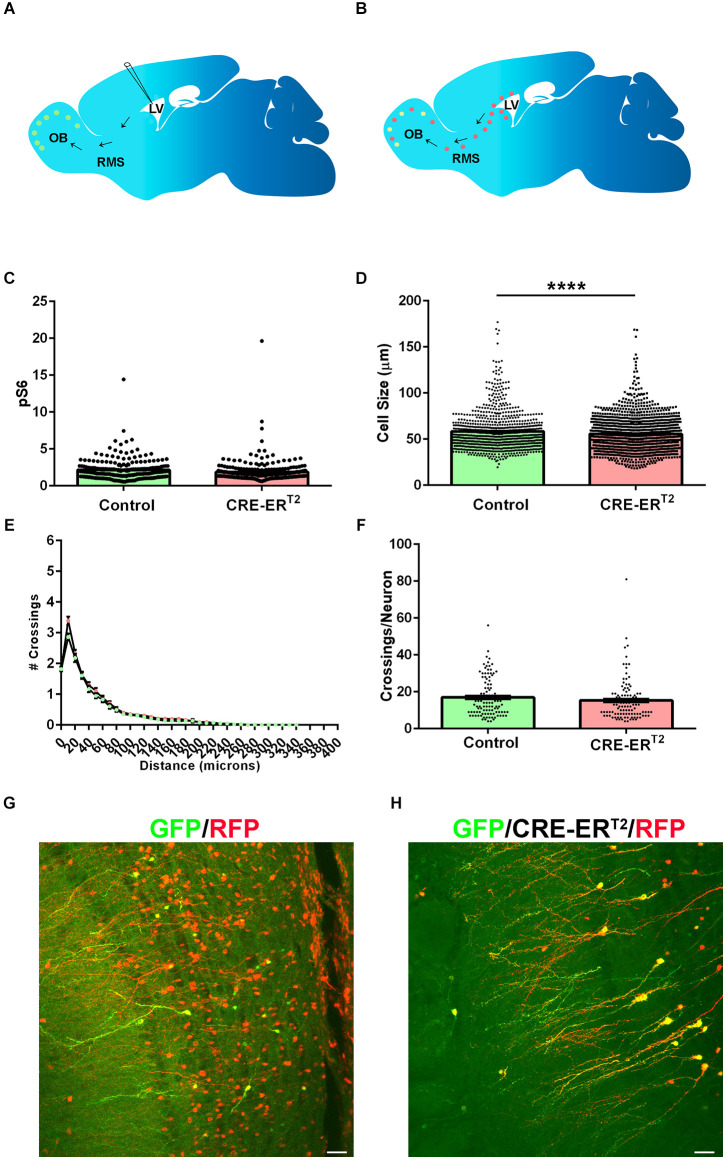
Loss of Tsc2 in mature GCs has modest effects on morphology. **(A)** Schematic diagram of neonatal electroporation and presence of GFP OB GCs prior to recombination at P30. **(B)** Schematic diagram of neonatal electroporation with the presence of GFP and RFP positive OB GCs at P60 after P30 tamoxifen induced recombination. **(C)** Quantification of pS6. **(D)** Quantification of cell size. **(E)** Sholl analysis. **(F)** Quantification of the number of dendrite crossings per GC. **(G)** RFP (red) and GFP (green) cells in OB GCs in control conditions. **(H)** RFP (red) and GFP (green) cells in OB GCs in CRE-ER^T2^ conditions confirm recombination. ****p < 0.0001.

The previous results demonstrate that *Tsc2* is required for GC development. It has been demonstrated that loss of *Tsc1* in cortical neurons causes similar morphological changes which can be reversed by mTORC1 inhibition, but only during a specific window of time. Based on these results and that deletion of Tsc genes from neurons results in morphological phenotypes, it is tempting to speculate that morphological plasticity might not exist for mature neurons. To answer whether loss of *Tsc2* from mature GCs alters maturation, neonatal electroporation of GFP and CRE-ER^T2^ or Tomato was performed at P0 (Figure 7A). Plasmid DNA is diluted by dividing NSCs. *Tsc2* was then conditionally deleted at P30 in mature GCs (GFP positive) or NSCs (GFP negative) by applying tamoxifen (Figure 7B). Mice were sacrificed and brains analyzed at P60. This allows the simultaneous labeling of two GC populations in each brain. GFP/RFP double positive GCs from CRE-ER^T2^ electroporations represent GCs having lost *Tsc2* at P30. In comparison, GFP negative RFP positive GCs are later born GCs that developed from NSCs that lost *Tsc2* at P30. As a control, GFP/RFP double positive GCs from GFP and Tomato electroporations represent GCs retaining *Tsc2*. mTORC1 was not significantly different in Tsc2^mut/mut^ GCs in comparison to *Tsc2^wt/wt^* GCs (*Tsc2*^wt/wt^, *n* = 228 cells, *N* = 4 mice, Mean = 2.155 ± 0.09378, *Tsc2*^mut/mut^, *n* = 236 cells, *N* = 4 mice, Mean = 1.889 ± 0.0.1010, *P* = 0.0548; Figure 7C). However, *Tsc2^mut/mut^* GCs were slightly larger (5%) in comparison to *Tsc2^wt/^*^wt^ GCs (*Tsc2*^wt/wt^, *n* = 881 cells, *N* = 4 mice, Mean = 58.34 ± 0.6904, *Tsc2*^mut/mut^, *n* = 1,341 cells, *N* = 4 mice, Mean = 55.56 ± 0.5082, *P* = 0.0010; Figure 7D). However, *Tsc2^mut/mut^* GC basal dendrites were not hypertrophic or extensively arborized compared to *Tsc2^wt/wt^* GCs at this age (*Tsc2*^wt/wt^, *n* = 110 cells, *N* = 4 mice, Mean = 149.4 ± 10.97, *Tsc2*^mut/mut^, *n* = 110 cells, *N* = 4 mice, Mean = 168.8 ± 9.743, *P* = 0.1867; Figures 7E,F). RFP and GFP co-expression confirmed electroporation in control OB GCs (Figure 7G) and recombination in CRE-ER^T2^ electroporated cells (Figure 7H). Taken together, while *Tsc2* is required to maintain GC mTORC1 activity and is indispensable for maturation, *Tsc2* is largely dispensable for the maintenance of mature GC morphology.

### RNA sequencing reveals mechanistic insight into neural circuitry

**Figure 8:**
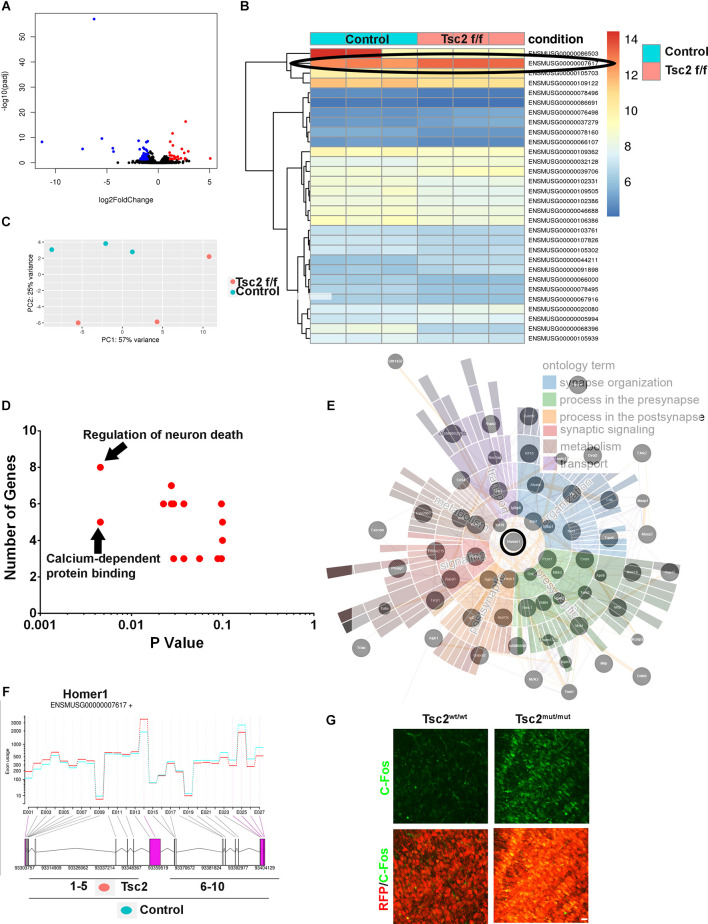
RNA sequencing reveals mechanistic insight into neural circuitry. **(A)** Volcano plot of RNA-seq of Tsc2^wt/wt^ and Tsc2^f/f^ OBs. **(B)** Heat map of RNA-seq data from Tsc2^wt/wt^ and Tsc2^f/f^. **(C)** PCA analysis of Tsc2^wt/wt^ and Tsc2^f/f^ OB RNA-seq data with (ENSMUSG000000007617=Homer1) circled. **(D)** GO terms listed based on P-values and the total number of transcripts represented in terms are differentially represented. **(E)** Network analysis of differentially expressed transcripts with Homer1 circled and SynGo terms overlapping network nodes. **(F)** Differential exon use mapping indicating Homer1a RNA transcripts is increased following Tsc2 deletion compared to controls. **(G)** C-Fos staining in Tsc2^wt/wt^ or Tsc2^f/f^ OB GC layer.

Transcriptome profiling was performed by sequencing RNA from OBs of* Nestin*-CRE-ER^T2^ mice that had been injected with tamoxifen at P0 and sacrificed at P60 to gain further mechanistic insight into the effect that losing *Tsc2* has on GCs. Volcano plots demonstrated groups of significantly differently expressed transcripts (Figure 8A). Significantly upregulated and downregulated transcripts following loss of *Tsc2* were visualized with heat maps (Figure 8B). Principal component analysis and bi-clustering of the top significantly regulated transcripts further revealed sample clustering. In all, 1,208 transcripts were differentially expressed (*p* < 0.05) or 99 with a p adjusted value less than 0.05 (Figure 8C). Network analysis of 51 genes that were significantly differentially expressed (p adjusted < 0.01) indicated that the gene ontology terms calcium signaling and neuron survival were significantly enriched (*p* < 0.05; Figure 8D). This same set of transcripts was used to search synaptic gene ontologies (SynGO; Figure 8E) which demonstrated potential changes in synapse organization, metabolism, transport, synaptic signaling, and processes in presynaptic and postsynaptic structure. This same set of transcripts was used in network analysis to identify nodes and node interactions that might provide insight into how OB GCs are altered (Figure 8E). At the core of this network was the mGluR coupling protein Homer1 which was also among the most highly expressed and significantly increased transcripts (Figures 8B,E). mGluR mediated long-term depression is altered in several TSC models (Auerbach et al., 2011). Homer1a is an immediate early gene splice form, the expression of which regulates neuron morphology and activity. Indeed, the Homer1a splice form was the topmost significantly enriched differentially spliced gene following loss of *Tsc2* (Figure 8F). C-Fos is an immediate early gene increased by neuronal activity (Sheng and Greenberg, 1990; Guthrie et al., 1993). Indeed, C-Fos staining was increased in OB GCs following loss of *Tsc2* (Figure 8G). Taken together, loss of *Tsc2* alters OB GC mTORC1 activity, soma size, dendrite arborization, and neural circuitry.

## Discussion

Here we demonstrate that *Tsc2* mutation disrupts the acquisition of GC characteristics. The results demonstrate that global and focal *Tsc2* deletion generates cytomegalic neurons with supraphysiological mTORC1 signaling that recapitulates key hallmarks of neurological lesions in TSC. Two genetic approaches were taken to achieve *Tsc2* deletion. The first approach relied on global *Tsc2* deletion from Nestin expressing cells by using the *Nestin*-CRE-ER^T2^ mouse which results in deletion from SVZ NSCs and daughter cells including neuroblasts (Lagace et al., 2007). Recombination was validated by widespread RFP expression and PCR for *Tsc2* recombined alleles in OBs. Recombination was further confirmed by the fact that mTORC1 activity was increased.

A complementary approach to delete *Tsc2* was taken to assess morphological changes. CRE electroporation into the neonatal SVZ was performed to achieve this goal. This technique has the benefit of mimicking somatic mosaicism seen with biallelic inactivation in TSC patients. RFP expression was detected ipsilateral to CRE and GFP expression indicative of recombination. OB images demonstrated that removal of *Tsc2* was associated with a disorganized distribution within the GC layer. Clusters of ectopically positioned cytomegalic GCs comprised most of these formations. Occasional dysmorphic neurons or cells with a tapered soma having an enlarged apical process proximal to the soma and an enlarged process projecting basally toward the RMS were identified. The release of reelin from Rheb over-expressing or *Tsc2*-null neurons and a reduced migration rate of neuroblasts are possible mechanisms responsible for the disorganization (Feliciano et al., 2012; Moon et al., 2015). The abnormal distribution of GCs is also reminiscent of the mislamination seen in cortical tubers (Mühlebner et al., 2016). However, mislamination is not required for seizures and network hyperexcitability induced in models of focal cortical dysplasia (Hsieh et al., 2016).

The appearance of cytomegalic neurons caused by mTOR activation has been unanimously reported for neurons including OB GCs. Indeed, mTORC1 hyperactivity was confirmed in *Tsc2* null OB GCs. Given that somatic mosaicism induced by electroporation, elevated pS6 was apparent in comparison to non-electroporated GCs, only following loss of both *Tsc2* alleles. In contrast, loss of one allele did not robustly or statistically significantly increase mTORC1. However, mTORC1 was detectable in GCs in control conditions and occasional *Tsc2* heterozygous cells had robust mTORC1 activity. It is unclear whether such cells in *Tsc2^f/wt^* mice might represent cells that have undergone loss of heterozygosity which is proposed to be sufficient for the formation of TSC lesions.

In support of mTORC1 activity being increased by loss of both TSC copies, *Tsc2^mut/mut^* GCs were on average larger than control cells at P30 and at P60. Although P60 *Tsc2^mut/mut^* GCs did not continue to grow between these ages, there were far more outliers at P60. This may represent two plausible scenarios. The first is that cell growth is permissible until P30, that there are growth promoting factors or an environment, be it nutrient related or biophysically malleable that is conducive to these events. *Tsc2* may therefore serve to control growth until P30. However, between P30 and P60, growth promoting cues might turn off. This would lead to no further function effect of loss of *Tsc2*. Alternatively, there may be additional constraints to growth such as the buildup of metabolites or depletion of ATP stores or nutrients. Indeed, metabolic rewiring is a feature of TSC mutant cells and metabolic stress due to mTORC1 regulation of autophagy is a key event in the loss of TSC neurons including in the OB. The fact that the average size of GCs did not increase in heterozygous cells further supports that loss of heterozygosity is necessary for manifestation of increases in soma size. However, there are statistical limitations to our analyses and to identifying the physiological significance of loss of one copy of *Tsc2*.

Neuroblasts take ~1 week to migrate from the SVZ through the RMS and empty into the OB (Petreanu and Alvarez-Buylla, 2002; Carleton et al., 2003). There, neuroblasts initiate radial migration into the GC layer. GCs begin to generate basal dendrites that form synapses with centrifugal fibers 10 days after being produced and receive GABAergic innervation within ~2 weeks (Belluzzi et al., 2003; Carleton et al., 2003). GC apical dendrites take ~2 weeks to project into the EPL but maturation of reciprocal synapses begins at ~3 weeks and receive glutamatergic innervation critical for activity dependent survival at ~4 weeks (Petreanu and Alvarez-Buylla, 2002; Belluzzi et al., 2003; Carleton et al., 2003).

Abnormal axon and dendrite morphology was reported in neurological disorders including in TSC. GCs are unique in that they are axonless yet release GABA from dendrites in the external plexiform layer of the OB. Extensive experimentation demonstrated that hyperactivation of mTOR increases OB GC dendrite growth and loss of mTOR pathway activity reduces dendrites. In support of the trophic effect of mTOR, loss of *Tsc2* increased the arborization, caliber, and number of dendrites. The increase in dendrite arborization occurred by P30 and continued to P60. Moreover, biallelic inactivation of *Tsc2* was required for the acquisition of hypertrophic dendrite arbors whereas no statistical difference was seen in *Tsc2* heterozygous cells. The study reported here cannot eliminate the possibility that *Tsc2* heterozygous GCs may have differences that are below the limit of detection of our instrumentation, that occur at a different time, or that are detected by increasing the number of specimens for statistical purposes. Nevertheless, experiments reported here, indicate that loss of heterozygosity is a critical determinant of dendrite morphology.

Neuron morphology was further assessed following removal of Tsc2 from mature GCs. Loss of *Tsc2* from mature GCs did not increase mTOR activity when compared to mature wild-type neurons. It is plausible that mTOR activation caused by loss of *Tsc2* in mature GCs may follow different temporal dynamics compared to GCs born without *Tsc2*. Thus, it is plausible that activation of mTOR from mature GCs occurred at earlier time points than would be expected or that hyperexcitability in the OB caused by deletion from NSCs might partially mask increases in mTOR activity. As a complementary readout of mTOR activity, soma size was measured. Mature GCs that lost *Tsc2* were modestly larger. This effect was minor in comparison to the increases in GC size seen following P0 *Tsc2* deletion. Incidentally, P30 and P60 wild-type GCs were of similar size to one another. These results indicate that growth of the soma largely takes place during a specific developmental period. It is unclear whether this period is caused by cell autonomous or environmental factors. However, it should be noted that soma growth of cortical neurons is similar in timing and that loss of *Tsc1* leads to changes in parvalbumin CINs and behavioral changes that occur during this same developmental window (Sokolov et al., 2018; Amegandjin et al., 2021). Taken together, our data point toward a critical developmental window for which *Tsc2* is required. The developmental window need not be thought about in relation to age, but rather in relation to the cellular state. While mTOR can be pushed by supraphysiological Rheb activity, this activity is carefully regulated by physiological signals and that *Tsc1/Tsc2* function as brakes. Thus, past a certain point of development, if signals to turn mTOR on are absent, loss of *Tsc1/Tsc2* may not have particularly dire consequences. Likewise, rapamycin during certain periods may be more effective at treating TSC phenotypes (Anderl et al., 2011; D’Gama et al., 2015; Cox et al., 2018). Recent reports also corroborate that loss of *Tsc1* has time developmental window specific effects on behavior in both glutamatergic and GABAergic neurons and in OB GCs (Amegandjin et al., 2021). In contrast, gain of mTOR activity in properly positioned mature neurons is sufficient to induce cortical hyperactivity, although abnormal placement of neurons is a key feature of TSC and focal cortical dysplasia (Hsieh et al., 2016). This has been rigorously tested by deleting *Tsc1* in 12–20 week old neurons using tamoxifen inducible Camk2-CRE-ER^T2^ × conditional *Tsc1* mice (Koene et al., 2019). Likewise, *in utero* electroporation of inducible mutant Rheb into NSCs followed by a dilution period allows for induction of Rheb expression in neuroblasts and neurons. Indeed, Rheb expression at P6 activates mTORC1 and induces seizures in these mice (Hsieh et al., 2016). Nevertheless, further experiments are required to more narrowly define the precise developmental windows that *Tsc2* genes are required for different cell types.

In addition to the major observation that loss of heterozygosity was necessary to identify detectable changes in this study, it should be noted that somatic mutations produced profound phenotypes that were dependent on the timing of induction. Thus, loss of TSC genes at different time periods could lead to variable outcomes. Incidentally, the modest changes that we identified in transgenic mice were overshadowed by the changes following electroporation. Given that there appear to be environmental cues required to activate or inactivate mTOR at different periods, we propose that graded loss of TSC genes during development could have unpredictable effects. For example, loss of *Tsc2* from most GCs might deplete the environment of factors or increase factors in the environment that limit phenotype. In the case of somatic mutations of the mTOR pathway, fewer cells affected could result in a more severe phenotype.

The OB is highly enriched in GCs. Therefore, a benefit of neonatal and global targeting of GCs is that transcriptomics studies are feasible. Previous studies performed on *Tsc1* knockout OB GCs identified that increases in FLNA are a key hallmark of TSC and underlie epileptogenesis (Zhang et al., 2014, 2020). Likewise, Hif-1a activity was demonstrated to be increased in *Tsc1* knockout OB GCs and is required for GC dendrite morphology and survival (Feliciano et al., 2012; Zhang et al., 2016). In the current study, RNA-sequencing unbiasedly identified numerous differentially expressed transcripts involved in processes including neuron survival and calcium signaling. Among the most notable increases was that of the activity dependent expression of Homer1. Homer1 is a ~100 kb gene that is subject to differential splicing (Bottai et al., 2002). While the Homer1b/c isoforms are constitutively expressed, Homer1a splicing is activity dependent and Homer1 is an immediate early gene (Bottai et al., 2002). Homer1 isoforms are localized to excitatory postsynaptic densities and linked to group 1 metabotropic glutamate receptors (Brakeman et al., 1997). Homer1a however is unique in that binding to mGluR causes a conformation change and constitutive mGluR activation even in the absence of a ligand (Tu et al., 1998; Xiao et al., 1998). Homer1a expression can modulate both neuronal activity and dendrite spine morphogenesis (Sala et al., 2003). Homer1a also regulates synaptic scaling analogous to LTD to decrease AMPA receptor activity (Hu et al., 2010). C-Fos also increased. Therefore, it appears that neural activity is increased in OB GCs following *Tsc2* loss. The results presented here are reminiscent of how neural activity is modulated in the hippocampus following loss of *Tsc1* (Bateup et al., 2011, 2013). Given that increased neural activity causes changes in activity dependent gene expression, it remains to be seen whether the changes identified in the OB represent drivers of abnormal activity or results of activity. Since Homer1a is predicted to reduce AMPA receptors, it seems more likely that the changes identified here reflect the increased activity of OB GCs. However further work is needed to understand the pathophysiological significance.

OB GCs are a heterogenous group that express subtype specific marker proteins, exhibit unique characteristics, and have different functions (Merkle et al., 2014; Nagayama et al., 2014; Takahashi et al., 2018). The measurements presented in our study are of GCs in the granule cell layers. Individual data points in graphs demonstrate a shift in the entire population analyzed which supports that Tsc2 is a conserved requirement across GCs. This general requirement is further supported by data from Nestin-CRE-ERT2 mice and from previous reports examining Tsc1-Rheb-mTORC1 signaling in OB GCs (Feliciano et al., 2012, 2013b; Lafourcade et al., 2013; Skalecka et al., 2016; Zhang et al., 2016; Sokolov et al., 2020). Cortical inhibitory neuron *Tsc1* deletion caused a population of somatostatin positive cells to adopt parvalbumin positive cells characteristics in a rapamycin dependent manner and demonstrates that there can be cell type specific effects caused by excessive mTORC1 signaling (Malik et al., 2019). However, future work is needed to determine whether there are OB GC subtype requirements and changes to characteristics.

Taken together, the results presented herein support the hypothesis that *Tsc2* is required for neural development and that loss of *Tsc2* results in abnormal biochemical, molecular, and developmental characteristics of inhibitory interneurons which may have relevance for understanding TSC neurological and neuropsychiatric manifestations.

## Data Availability Statement

The datasets presented in this study can be found in online repositories. The name of the repository and accession number can be found below: National Center for Biotechnology Information (NCBI) Gene Expression Omnibus (GEO), https://www.ncbi.nlm.nih.gov/geo/, GSE206287.

## Ethics Statement

The animal study was reviewed and approved by Clemson University Institutional Animal Care and Use Committee.

## Author Contributions

Conceptualization, supervision, and funding acquisition: DF. Methodology, validation, and} investigation: DF, JH, VR, and AS. Formal analysis, writing—original draft, writing—review and editing, visualization: DF, JH, and VR. Project administration: DF and JH. All authors contributed to the article and approved the submitted version.

## Funding

DF is supported by United States of America Department of Defense U.S. Army Medical Research Activity Award Congressionally Directed Medical Research Program Tuberous Sclerosis Complex Research Program W81XWH2010447, National Institutes of Health R15NS096562 and 5P20GM139769-03.

## Conflict of Interest

The authors declare that the research was conducted in the absence of any commercial or financial relationships that could be construed as a potential conflict of interest.

## Publisher’s Note

All claims expressed in this article are solely those of the authors and do not necessarily represent those of their affiliated organizations, or those of the publisher, the editors and the reviewers. Any product that may be evaluated in this article, or claim that may be made by its manufacturer, is not guaranteed or endorsed by the publisher.
